# On Dreams and Motivation: Comparison of Freud’s and Hobson’s Views

**DOI:** 10.3389/fpsyg.2016.02001

**Published:** 2017-01-06

**Authors:** Simon Boag

**Affiliations:** Department of Psychology, Macquarie University, SydneyNSW, Australia

**Keywords:** disguise-censorship, dreams, fantasy, motivation, psychoanalysis, neuropsychoanalysis, repression

## Abstract

The merits of Freudian dream theory continue to be debated and both supporters and critics appeal to empirical evidence to support their respective positions. What receives much less attention is the theoretical coherency of either Freudian dream theory or alternative perspectives. This paper examines Freudian dream theory and J. Allan Hobson’s alternative position by addressing the role of motivation in dreams. This paper first discusses motivation in Freudian theory and its relation to dreams and disguise-censorship. The role of motivation in Hobson’s theory is then considered. Hobson’s claim that dream plot and content selection is random and based on design error and functional imbalance is then discussed in relation to the protoconsciousness theory proposal that dreams serve an adaptive function. While there are apparent inconsistencies in Hobson’s position, his appeal to emotions and instincts provides a preliminary platform for understanding the role of motivation in dreams that is consonant with the Freudian position.

## Introduction

“Psychoanalysis is founded upon the analysis of dreams...”

(Freud, 1912, p. 265).

While psychoanalytic approaches have more or less changed since Freud, one area of Freudian theory that continues to draw attention and debate is his theory of dreams (Solms, 2000a, 2013a; Hobson, 2013, 2015; Erdelyi, 2014; Colace and Boag, 2015a,b). For Freud, dreams express the hallucinatory fulfillment of wishes, and while this claim was empirically informed, Freud also believed that such a claim was logically necessitated. In Freud’s view, all mental activity—including dreaming—is motivated by endogenous stimuli (*Triebe*, or the ‘drives’—Freud, 1905, 1915). Such motivational drives are the somatic engines that provide the motivational policy of cognition with respect to the urgency and content of desires, fantasy, and affective states (Maze, 1983). At the same time, Freud further recognizes that sleep is biologically necessary and he postulates that dreams serve the function of acting as the guardians of sleep: when a drive state arises during the night and threatens to interrupt sleep, the dreamt hallucinatory experience of satisfaction allows sleep to continue. Freud accordingly writes, “[s]ince a dream that shows a wish as fulfilled is *believed* during sleep, it does away with the wish and makes sleep possible” (Freud, 1901, p. 678, his italics).

Aserinsky and Kleitman’s (1953) discovery that dreams typically occur during rapid eye movement (REM) sleep, however, provided a foundation for later critics to declare that Freud’s wish-fulfilling dream theory was clearly wrong (Hobson and McCarley, 1977; McCarley and Hobson, 1977). Instead, it was supposed, REM dreaming sleep is instigated by brain stem activation, and dream bizarreness is explicable in terms of random ponto-geniculo-occipital (PGO) brainstem activation, aminergic demodulation, and deactivation of the dorsolateral prefrontal cortex (Hobson and McCarley, 1977; Hobson, 2007, 2009, 2013). On this view, dreams are “bizarre because of the loss of the organizing capacity of the brain, not because of an elaborate disguise mechanism that rids an internal stimulus of an unacceptable meaning …” (Hobson and Pace-Schott, 1999, p. 211), and, if anything, dreams tend to be emotionally transparent (Hobson, 2009, 2013). Hobson (2013) consequently concludes that Freudian dream theory is “obsolete and entirely replaceable” (p. 144; cf. Hobson, 2014a,b,c,d).

Nevertheless, the evidence of a double-dissociation between REM sleep and dreaming (*viz*. dreams can occur without REM and REM without dreams) provides a “body blow to the REM-dreaming connection” (Erdelyi, 2014, p. 115; cf. Solms, 2000a; Colace, 2003; Bischof and Bassetti, 2004; Domhoff, 2005). Furthermore, while Hobson presents his position as antithetical to the Freudian one, more than one commentator has noted that Hobson’s position is increasingly converging with the Freudian one in several respects (Domhoff, 2005; Boag, 2006b; Kessler, 2013; Solms, 2013a; Colace and Boag, 2015b; Hopkins, 2016). For instance, there is more than a passing semblance between Hobson’s theory and Freud’s (1900) account of dreams as hallucinatory primary process (Kessler, 2013; Solms, 2013a; Hopkins, 2016). More fundamentally, however, Hobson’s initial claim that dreams are “motivationally neutral” (McCarley and Hobson, 1977, p. 1219), has been tempered with the frank concession that “the unfettered play of dopamine in REM sleep is in keeping with the assumption that dreaming is “motivated” and that important motivational goals may be revealed in dreams” (Hobson, 2014c, p. 41). The significance of this concession is difficult to overstate. Dopamine and the mesocortical/mesolimbic dopaminergic system have been consistently linked to motivational states and goal-directed behavior, including appetitive interactions (‘wanting’) and hedonic reward (Berridge and Robinson, 1998; Solms, 2000a; Berridge, 2004, 2007; Colace, 2004; Alcaro et al., 2007; Dahan et al., 2007). While there may be alternative explanations of the role of dopamine in dreaming (Perogamvros et al., 2013), Hobson’s acceptance of the role of both dopamine and motivation in dreams appears to be endorsing Freud’s fundamental postulate about the motivated nature of dreaming, leading Solms (2013a, p. 205) to comment that “Hobson appears to be tentatively coming around to the Freudian view” (cf. Colace and Boag, 2015b).

Since motivation appears to be relevant to understanding dreams, some consideration of motivational theories within the respective accounts provides one method for addressing where the fundamental differences lie between Freud’s and Hobson’s theory and for considering the adequacy of either position. The aim of this paper is to assess the accounts of motivation within both Freudian dream theory and Hobson’s alternative position and determine the precise points of disagreement. To achieve this, the paper first discusses the logical requirements for a coherent theory of motivation. Freud’s theory of dreams is then discussed and two distinct positions in Freudian theory with respect to disguise-censorship are identified. Freud’s ‘censor’ of dreams is rejected on logical grounds and an alternative role for ‘censorship’ is proposed. Hobson’s alternative dream account is then similarly reviewed and the role of motivation evaluated. Hobson’s claim that dream plot and content selection is random and based on design error and functional imbalance is then discussed in relation to his hypothesis that REM sleep dreaming is adaptive.

## Freud’S Theory of Motivation and Action

At its broadest, motivation entails what *moves* us (Boag, 2017), and motivational accounts address why we seek out certain things at specific times and why we have varying responses to the same stimuli (Berridge, 2004; Wise, 2004). For this reason, “[a]ll behavior, with the possible exception of simple reflex actions, requires motivation” (Sewards and Sewards, 2003, p. 25) and thus motivation is a necessary consideration for any explanation of what we do^1^. While psychoanalytic theories of motivation are diverse (see Boag, 2017), the Freudian position developed here proposes a physiologically based theory of motivation which is consistent with a Darwinian evolutionary perspective and comprehensible within a natural science framework (see Maze, 1983). One way of appreciating Freudian motivation theory is with respect to desires and beliefs. In the simple desire-belief model, ‘purposive’ behavior can be understood in terms of desires and beliefs acting as causal antecedents. As Wollheim (1991) and others appreciate (e.g., Petocz, 1999, 2015; Boag, 2012, 2016; Pataki, 2014, 2015), Freud both extended and deepened the common-sense desire-belief account. The ‘desire’ component provides the motivational policy for action while the ‘belief’ component (which includes knowing, believing, planning, etc.) plays an instrumental role with respect to guiding the desire. Taken as such, explaining person *P*’s doing *A* involves: (i) *P* desiring *B*; and (ii) *P* believing that doing *A* leads to *B*. An act of drinking, for instance, is explicable with respect to both a desire to drink and a belief that water will satisfy that desire (Wollheim, 1991). Beliefs, however, while necessary, are not sufficient for explaining behavior since they are *policy neutral* and cannot explain why one person acts upon the belief and why another person does not (e.g., Maze, 1983, 1987; Michell, 1988; Mackay, 1996; Boag, 2012, 2016). The same belief may or may not be acted upon, depending upon what one desires (Maze, 1987), and so cognition alone cannot be the driver of action (see Boag, 2012, 2017, for further discussion).

The topic of mental causation has a long and complex history (see, for example, Kim, 2000) and appealing to ‘desires’ as efficient causes is problematic since desires themselves entail a person desiring some state of affairs, and, so considered, are cognitive acts (where *S* desires *p*). While psychological acts themselves may be causally efficacious, using desires as explanations of actions conflates causes and effects since there is no clear distinction between the desire (as *explanans*) and the behavior (as *explanandum*) (*viz*. if we try to explain *S* doing *p* by postulating a ‘desire for *p*’ then the effect is logically connected to the cause and thus cause and effect are conflated with one another, and so no real explanation is provided—Maze, 1983, pp. 24–25). Instead, causes and effects must be logically distinct to avoid the circularity associated with explaining the effect in terms of itself (see Maze, 1983; Boag, 2011, 2015b). Since a ‘desire for *x*’ describes a relationship (what the person is doing, *viz*. desiring), we essentially need to know about the conditions giving rise to desire in the first place, independently of the effect (i.e., the structures and interactions giving rise to that desiring relationship—Maze, 1983). As Mackay (1996, pp. 10–11) points out, “any theory must, if it is to be coherent and a serious account of motivation, specify the states of the person that motivate action independently of the motivated action, and be able to show in principle how such states become linked to objects.”

One of Freud’s (1905, 1915) contribution here is to substantiate the motivational foundations of desires in the form of drives as somatic motivational systems. On this view, the organism is pressured from both stimuli that impinge from the external environment and stimuli that arise internally, in the shape of somatic drives (*Triebe*). These drives as ‘biological engines’ provide the embodied motivational foundations driving thought and behavior, and determine the innate policy of human activity, even if their aims are modifiable via experiences (see Maze, 1983). Freud (1910) initially grouped these drives according to self-preservation (e.g., hunger) or libidinality (e.g., sexuality), and later between life and death instincts (Freud, 1920). While the death drive lacks any convincing evidence, the self-preservative and libidinal drives (e.g., hunger and sexuality) appear fairly uncontroversial empirically (e.g., Berridge, 2004; see also Bazan and Detandt, 2013 for a comparison of contemporary neuroscience and Freudian drive theory) even if possibly not the whole story (i.e., both homeostatic drives and primary emotional systems require consideration—Watt, 2008; Solms and Zellner, 2012; see Boag, 2017 for further discussion). The role of the dopaminergic SEEKING system proposed in affective neuroscience is also especially relevant here (e.g., Panksepp, 1999; Panksepp and Biven, 2012). The SEEKING system is involved in the instigation of goal-seeking behaviors and appetitive interactions with the world (Panksepp, 1999; Panksepp and Biven, 2012), and given both its connection to motivation and activity during dreaming (Solms and Turnbull, 2002; Colace, 2014; Colace and Boag, 2015a), any comprehensive account of dreaming must account for this motivational component.

For Freud (1915), somatically based drives are the policy makers of desires and affective responses generally, whereas affects appear to be the drives’ embodied experience of their relations toward objects (Boag, 2008, 2012, 2014). Such primary drive sources are logically independent of their effects and so avoid circular explanation. Freud (1915) further recognizes that the somatic component of drives is also necessary for avoiding vacuous claims about the nature and number of primary drives (see also Maze, 1983). However, Freud’s assumption that behavior and cognitive activity involve drives discharging energy (the so-called ‘drive-discharge’ theory) is problematic since there is no evidence of any such accumulation and discharge of energy (see Bowlby, 1969; see also Boag, 2017). Nevertheless, the drive concept is logically necessitated for any satisfactory account of explaining why humans do what they do (see Maze, 1983) and can be salvaged by dispensing with discharge processes and instead positing feedback systems (see Maze, 1983; Boag, 2017 for extensive discussion).

Rather than divorcing the bodily ‘passions’ from the ‘rational faculty’ of the mind, in Freud’s view both mind and body, as well as cognition and motivation, are intimately linked (Freud, 1915). For Freud, the drives are *psychobiological* systems: drives typically require interactions with the external environment for their gratification—to satisfy hunger, for example, requires finding and eating food. Freud’s view is consistent with research linking both drives and emotional systems with cognitive states (e.g., Watt, 2012, p. 100; Wright and Panksepp, 2012, p. 24; see Boag, 2017 for further discussion). Cognition guides motivated behavior in that organisms interact and learn about the environment, fundamentally with respect to what is satisfying or frustrating. Freud outlines this in his hungry baby example when he describes the development of wishes:

The exigencies of life confront it (the organism) first in the form of the major somatic needs. The excitations produced by internal needs seek discharge in movement, which may be described as an ‘internal change’ or an ‘expression of emotion.’ A hungry baby screams or kicks helplessly. But the situation remains unaltered, for the excitation arising from an internal need is not due to a force producing a *momentary* impact but to one which is in continuous operation. A change can only come about if in some way or other (in the case of the baby, through outside help) an ‘experience of satisfaction’ can be achieved which puts an end to the internal stimulus. An essential component of this experience of satisfaction is a particular perception (that of nourishment, in our example) the mnemic image of which remains associated thenceforward with the memory trace of the excitation produced by the need. As a result of the link that has thus been established, next time this need arises a psychical impulse will at once emerge which will seek to re-cathect the mnemic image of the perception and to re-evoke the perception itself, that is to say, to re-establish the situation of the original satisfaction. An impulse of this kind is what we call a wish; the reappearance of the perception is the fulfillment of the wish... (pp. 565–566, Freud’s italics).

A wish then is a desire to re-experience a state of satisfaction experienced previously, when the need, previously satisfied, re-appears. The ‘desire for’ the remembered satisfaction leads to the formation of the wish, where the wished-for situation may be either for some state of affairs to occur, or some other state of affairs not to occur (Mackay, 1996). Given the somatic-motivational connection to wishes, should a wish remain unsatisfied then the organism will remain in a state of desire (and associated frustration) unless an alteration to the motivating conditions is achieved. Such alteration is more or less possible with respect to securing secondary aims if the primary aim is denied (i.e., finding so-called substitute objects). On the other hand, wish-fulfilling hallucinatory satisfaction may also, at least temporarily, pacify the need. Here Hopkins (1995) proposes a distinction between drive *pacification* and *satiation*. A drive system may be more or less temporarily pacified if the wished-for situation is believed to obtain (through illusory gratification). On the other hand, *satiation* occurs when the actual satisfying conditions necessary for terminating a drive are obtained. As Freud (1900, 1940) recognizes, pacification via hallucinatory wish-fulfillment is ultimately futile in the longer term, and the individual is more or less forced to pay attention to reality for satiating drive states.

‘Thought’ generally involves motivated engagement with objects (i.e., is wishful), and according to Freud, the content of thought is related to motivation rather than occurring as a matter of chance. Freud further recognizes that the so-called reality principle is but a modification of the basic motivational position (the so-called unpleasure-pleasure principle—Freud, 1911, p. 219; see also Boag, 2015a). Thoughtful activities such as ‘prediction’ and ‘expectation’ are associated with the mesocorticolimbic dopamine system (Solms, 2013a), which is congruent with a motivated drive account (cf. Phillips et al., 2003; Brooks and Berns, 2013). Freud accordingly writes that “all this activity of thought merely constitutes a roundabout path to wish-fulfillment which has been made necessary through experience. Thought is after all nothing but a substitute for a hallucinatory wish” (Freud, 1900, p. 567). To propose otherwise would be to posit a mind independent of its somatic-motivational foundation—a mind existing as a veritable disembodied Cartesian rational faculty—and such an account fails to address the necessary role of the body and its motivational engines for understanding cognitive activity (Maze, 1983, 1987; Boag, 2014, 2017 ^2^). Freud’s major thesis that dreams act as the fulfillment of wishes thus needs to be understood within the context of his greater motivational theory.

## Freud’S Theory of Dreams and the Role of Motivation

Consistent with Freud’s motivational account, dream content is causally related to the motivated-affective life of the organism, no less than any other ordinary mental process. Freud writes that “there is, of course, no such thing as arbitrary determination in the mind” (Freud, 1901, p. 680; cf. Freud, 1900, p. 514), which naturally extends to dreams: “we regard nothing in a dream as accidental or indifferent...” (Freud, 1916–1917, p. 119). While organic sensations (such as digestive disturbances) may make some contribution to dream content, dreams do not originate in bodily sensations and such sensations are also not sufficient for explaining dreaming activity since they lack any motivational foundation (see Freud, 1900, pp. 220ff). Instead, Freud believes that a ‘wish’ is necessary for dream activity because it provides the *motive force* for it (e.g., Freud, 1900, pp. 487, his italics, 560–561; Freud, 1916–1917, p. 226): “it is self-evident that dreams must be wish-fulfillments, since nothing but a wish can set our mental apparatus at work” (Freud, 1900, p. 567). The term ‘force’ is open to interpretation but since dreaming involves attending to some objects and events and not others, Freud is essentially proposing that motivation is the key for explaining why someone dreams of *x* while ignoring *y*^3^.

While Freud (1900) believes that dreams are the expression of wishes, not all wishes are equally acceptable and some become censored and distorted via repression. As a result of this censorship, both the objectionable content and the dream’s wish-fulfilling character become obscured. This censorship allows the otherwise objectionable content to be expressed, while protecting the dreamer from anxiety. However, certain children’s dreams provide good examples of transparent wish-fulfilling dreams, and such dreams may also be seen in adults under certain conditions such as deprivation, where, for example, starving people report dreaming frequently of food, or even less drastically, following mild dehydration after eating salty anchovies, such as occurs in Freud’s dream of drinking water (Freud, 1900, pp. 123–124). Similarly, the frequency of drug dreams during withdrawal provides further evidence of transparent wish-fulfillment, consistent with Freud’s drive account (see Johnson, 2001; Colace, 2010, 2012, 2014; Colace and Boag, 2015a). In respect to both the role of the dopamine pathways in dreaming, and the relationship between dopamine, motivation, and desire referred to earlier (Berridge, 2004; Alcaro et al., 2007; Dahan et al., 2007), the evidence if anything, appears to support Freudian theory (see Colace, 2014), a point that Hobson (2014c, 2015) appears to more or less concede.

## Anxiety Dreams

Anxiety dreams, however, appear to provide *prima facie* evidence against the claim that dreams are wish-fulfilling, and Hobson (2014d, p. 70) believes that “Freud never came to terms with the fact of dream anxiety” (cf. Hobson, 2007). Freud was, of course, well aware of anxiety dreams and their implications for his theory, beginning his chapter on ‘dream distortion’ in the *Interpretation of Dreams* on precisely this point (Freud, 1900, pp. 134ff). Therein Freud addresses anxiety and dream distortion in terms of conflicting aims, repression, and disguised fulfillment of wishes. More generally, Freud (1900, Freud, 1916–1917) explains dream bizarreness—the unusual, improbable and even impossible characteristics of dreams compared to waking experience—in terms of both primary process mentation (processes such as displacement and condensation) and ‘disguise-censorship.’

Freud addresses anxiety dreams in terms of motivational conflict. In Freud’s (1915, 1940) account, the organism is motivated by a number of drives and so the mind is pictured as an economy of competing motives that can sharply conflict with one another. Conflict, in this context, is typically understood to be between ‘improper’ wishes and the ethical or aesthetic ideals of the ego, and anxiety dreams are described as products of conflict: “Something that is a satisfaction for the unconscious id may for that very reason be a cause of anxiety for the ego” (Freud, 1940, pp. 170–171). In other words, the fulfillment of one wish might also represent the frustration of another, whereby, for example, a particular wish may be fulfilling sexually but be distressing ethically. Consider, for instance, Freud’s (1917) own dream reported in the *Uncanny*, where he finds himself in a red light district from which he tries to escape, but finds himself back there again, and again, despite his apparent wish to the contrary. Freud was all too aware of the interpretation of these dreams, and such ‘nightmares’ therefore do not refute the wish-fulfilling character of dreams since Freud’s theory does not assume that the dream is simply a gratifying experience for the dream-ego (Freud, 1900, Ch. 4; Freud, 1916–1917, pp. 215–216; Freud, 1940, pp. 170–171). As Freud writes, “the dreamer fighting against his own wishes is to be compared with a summation of two separate, though in some way intimately connected, people” (Freud, 1916–1917, pp. 218–219). And again:

No doubt a wish-fulfillment must bring pleasure; but the question then arises ‘To whom?’ To the person who has the wish, of course. But, as we know, a dreamer’s relation to his wishes is a quite peculiar one. He repudiates them and censors them—he has no liking for them, in short. So that their fulfillment will give him no pleasure, but just the opposite; and experience shows that this opposite appears in the form of anxiety, a fact which has still to be explained. Thus a dreamer in his relation to his dream-wishes can only be compared to an amalgamation of two separate people who are linked by some strong element in common (Freud, 1916–1917, pp. 215–216).

Accordingly, Hobson’s (2007, p. 1113) claim that “Freud was aware of the inability of his dream theory to account for the negative emotions of many dreams” lacks any substantive basis. Nevertheless, anxiety dreams are not, in and of themselves, bizarre (in the sense of exhibiting fantastical and impossible events), although they might appear strange and alien within the context of the dreamer’s ethical self-view. Furthermore, such dreams do not actually reflect repression *per se* since, if anything, anxiety dreams indicate a relative absence of repression (see Colace et al., 2015 for a case study consistent with this position).

## Censorship and Dream Distortion

While anxiety dreams indicate a relative absence of repression, Freud believes that intolerably distressing content may nevertheless be ‘censored.’ It is on this issue where a particular point of contrast arises between Freud’s and Hobson’s theory concerning whether ‘disguise-censorship’ contributes to dream-bizarreness (Hobson, 2007, 2013, 2015). Disguise-censorship generally refers to the position whereby dream bizarreness and distortion are attributable to “the psychic censor (that) acts to screen and block wishes unacceptable to consciousness” (McCarley and Hobson, 1977, p. 1218). According to Freud (1900, Freud, 1916–1917), during sleep, the repressing functions of the ego are reduced (though not completely inactivated) and repressed wishes continue to push for expression. Nevertheless, Freud believes that a censorship still exerts a pressure, leading to objectionable wishes becoming distorted and ‘disguised,’ in tandem with primary process mentation (Freud, 1916–1917, p. 149).

For Hobson, Freud’s disguise-censorship explanatory strategy is “the heart of Freudian dream theory” (Hobson, 1999, p. 170) and so to renounce disguise-censorship would be to renounce Freudian dream theory: “After all is said and done, disguise-censorship is closer to the heart of the Freud-Solms dream model than wish-fulfillment… The problem … is that if disguise-censorship is explicitly renounced … there is really nothing left to the Freudian dream theory” (Hobson and Pace-Schott, 1999, pp. 211–212). However, while critical of the censor account, Hobson does not rule out that repression may contribute to dream content, even if denying any essential role to the process:

Dynamically repressed (or actively forced down) mental content may well emerge in the process of dream image creation and plot selection processes that activation-synthesis credits with dream production, but such material is neither necessary nor sufficient for dreaming to occur in sleep (Hobson, 2014d, p. 69; cf. Hobson, 1988).

At the same time, disguise-censorship enjoys an uncertain status within psychoanalytic dream theory. Solms (2000b, p. 194), for instance, indicates that “the neuroscientific data do not seem to require the hypothesis of an active distorting agency,” whereas Solms and Turnbull (2002, p. 215) propose that regression to primary-process mentation alone may explain dream bizarreness, “with no need to introduce the additional function of censorship.” Nevertheless, if alternate accounts of repression exist without recourse to a ‘psychic censor,’ then repression’s role in dreaming requires fresh consideration.

The first problem with assessing Freud’s censorship account is that it is not at all clear what Freud actually means by ‘censorship.’ As developed elsewhere (Boag, 2006a, 2012), Freud typically uses the more general word ‘*Zensur*’ (censorship), although on rare occasions he uses ‘*Zensor*,’ the latter more easily interpreted as a little guard who watches over what becomes conscious. Freud, for instance, anthropomorphises this latter censor as a ‘watchman’: “there are occasions when that excellent fellow the night-watchman, whose business it is to guard the little township’s sleep, has no alternative but to sound the alarm and waken the sleeping townspeople” (Freud, 1940, p. 171). Taken literally, this censor is an active, cognizing agent independent of the ego (a homunculus of sorts), acting to guard the ego during sleep. This is the position explicitly targeted by Hobson:

According to PDT (psychoanalytic dream theory), dreaming occurs because unconscious infantile wishes, which are easily suppressed during waking, become active in sleep. When the ego is off duty, the id becomes unruly. To the rescue of the sleeping ego come the defensive forces of disguise and censorship. They bowdlerize the kinky id forces and make them look non-sensical and meaningless whereas, in fact, they are masquerades for viciously potent entities that would overwhelm consciousness if admitted to that realm undisguised (Hobson, 2014d, pp. 67–68).

Hobson’s view is not unjustified, since Freud does propose that this censor determines what may or may not become conscious:

We find that there is a ‘censorship,’ a testing agency, at work in us, which decides whether an idea cropping up in the mind shall be allowed to reach consciousness, and which, so far as lies with its power, ruthlessly excludes anything that might produce or revive unpleasure (Freud, 1913, pp. 170–171; cf. Freud, 1932, p. 221).

Furthermore, Freud also writes that this censor ‘disguises’ these content: “the second agency (censorship) allows nothing to pass without exercising its rights and making such modification as it thinks fit in the thought which is seeking admission to consciousness” (Freud, 1900, p. 144; cf. Freud, 1916–1917, p. 140). Similarly, in connection with dreams, the censor acts as an independent, cognizing agency, ever vigilant and on alert to protect the sleeping ego:

If we enter further into the structure of the ego, we may recognize in the ego ideal and in the dynamic utterances of conscience the *dream-censor* [*Zensor*] as well. If this censor is to some extent on the alert even during sleep, we can understand how it is that its suggested activity of self-observation and self-criticism... makes a contribution to the content of the dream (Freud, 1914, pp. 97–98).

This censor thus stands above the rest of the ego, such that whilst the ego succumbs to sleep, the censor remains vigilant, albeit in an attenuated state (see Boag, 2006a, 2012). It is precisely here where problems emerge for Freud’s account. Freud is invoking a strongly partitive account of mind by proposing multiple knowers within the personality. Strong partitioning, in itself, is not necessarily a fatal objection (see Boag, 2005), but since Freud’s censor is characterized solely by its effects, Freud appears to be engaging in an instance of reification (see Boag, 2005, 2012). Furthermore, the functional roles attributed to the censor are problematic. Taken literally, the censor is said to cognize and evaluate other mental processes (impulses and desires) before either allowing, forbidding or disguising these. If so, the censor must first detect and determine whether a wish is forbidden or acceptable, and know strategies for censoring and distorting these to minimize offense. Add to this the censor’s standing in a privileged position for exercising such duties, and it becomes easy to see why critics have seized upon this account of the censor as an omnipotent, transcendental agency (e.g., Bonanno and Keuler, 1998; cf. Boag, 2006a, 2012). Consequently, Hobson (2007; 2014b; 2014d) is justified in dismissing Freud’s anthropomorphic account of “the psychic censor (that) acts to screen and block wishes unacceptable to consciousness” (McCarley and Hobson, 1977, p. 1218).

## An Alternative Account of Repression in Dreaming

Censor accounts as found above have proliferated within psychoanalytic theorizing in one guise or another (see Boag, 2012), partly because such accounts appear necessary for explaining how repression could occur (see Maze and Henry, 1996; Boag, 2007a, 2012). Nevertheless, Freud also indicates that the ‘censor’ should not be taken literally or anthropomorphically: “I hope you do not take the term too anthropomorphically, and do not picture the ‘censor of dreams’ as a severe little manikin or a spirit living in a closet in the brain and there discharging his office; …. For the time being it is nothing more than a serviceable term for describing a dynamic relation” (Freud, 1916–1917, p. 140). And further:

We may here recall that we have found that the formation of dreams takes place under the dominance of a censorship [*Zensur*] which compels distortion of the dream-thoughts. We did not, however, picture this censorship as a special power, but chose the term to designate one side of the repressive trends that govern the ego, namely the side which is turned toward the dream-thoughts (Freud, 1914, pp. 97–98).

Taken as such, ‘censorship’ is a description of the outcome of repression, and so Freud must still provide an explicit account of how repression and ‘censorship’ then actually occur. An alternative position to the anthropomorphic censor views the act of repression itself as simply another drive-activity, akin to fleeing or avoiding aversive stimuli, rather than an activity exercised by a rational executive or higher function of the mind (see Boag, 2007b, 2012, 2017). Viewed in this manner, one way that repression could contribute to dream distortion is via the inhibition of primary aims and the development of substitute ones (see Boag, 2006a). In Freud’s view, while the aim of drives is satisfaction, the conditions and means necessary for satisfaction vary and a distinction between primary and secondary aims can be made (Freud, 1915, p. 122). The aims believed to be the most direct route to satisfaction are the primary aims or objects of the drive (cf. Petocz, 1999), and should these primary aims be associated with negative consequences and repressed out of anxiety, then substitute secondary aims may act as alternative, compromised routes to satisfaction. As Freud writes, “[t]he instinctual demands forced away from direct satisfaction are compelled to enter on new paths leading to substitutive satisfaction...” (Freud, 1940, p. 201; cf. Freud, 1920, p. 11). The repressed wish, in relation to the demands of anxiety, may become distorted and lose semblance from its primary objective.

Recently I have proposed, following lines of thought in Freud’s own writings that repression involves knowing the targets of repression, but preventing knowing (or acknowledging) that the repressed targets are known (Boag, 2008, 2012). On this account, repression reflects an apparent paradoxical state of affairs whereby a person can be said to both know the targets of repression, but also not know that the targets are known. Freud first describes such a state of affairs as ‘blindness of the seeing eye’ in the case of Miss Lucy R in the *Studies on Hysteria* (Breuer and Freud, 1895; see Boag, 2015a). Unlike ‘turning a blind eye,’ whereby a person knowingly ignores some undesired state of affairs, ‘blindness of the seeing eye’ involves both knowing and not knowing some distressing fact simultaneously. This view of repression does not require a censor deciding what can or cannot become conscious because the ego’s knowing the repressed generates anxiety which in turn prevents knowing that the repressed target is known. Such repression is presumably mediated via neural inhibition and addresses how apparently paradoxical processes might occur, including unconscious resistance (cf. Freud, 1909; see Boag, 2012) and anosognosia (see Boag, 2012). This position can be further extended to understanding phenomena such as ‘blindsight,’ which appear to similarly require such paradoxical unconscious knowing (cf. Weiskrantz, 1986; see Boag, 2017; see also Stevens, 2016, for a discussion of the possible role of the anterior cingulate cortex in repression)^4^.

To illustrate how such blindness of the seeing eye possibly contributes to dreaming, consider Freud’s celebrated dream of Irma’s injection. Freud explains that during the summer of 1895 he had been giving psychoanalytic therapy to a young woman named Irma. The treatment had ended “in a partial success; the patient was relieved of her hysterical anxiety but did not lose all her somatic symptoms” (Freud, 1900, p. 106). Freud then received a visit from Otto, a ‘junior colleague’ who had been staying with Irma, and Otto informed Freud that Irma was “better, but not quite well.” Freud writes:

I was conscious that my friend Otto’s words, or the tone in which he spoke them, annoyed me. I fancied I detected a reproof in them, such as to the effect that I had promised the patient too much; and, whether rightly or wrongly, I attributed the supposed fact of Otto’s siding against me to the influence of my patient’s relatives, who, as it seemed to me, had never looked with favor on the treatment (p. 106).

The same evening of this event, Freud wrote out Irma’s case history, with the idea of giving it to a common friend, Dr. M., “in order to justify myself” (p. 106). Freud was obviously perturbed by the insinuation, dreaming that same night:

A large hall—numerous guests, whom we were receiving.—Among them was Irma. I at once took her to one side, as though to answer her letter and to reproach her for not having accepted my ‘solution’ yet. I said to her: ‘If you still get pains, it’s really only your fault.’ She replied: ‘If you only knew what pains I’ve got now in my throat and stomach and abdomen—it’s choking me’—I was alarmed and looked at her. She looked pale and puffy. I thought to myself that after all I must be missing some organic trouble. I took her to the window and looked down her throat, and she showed signs of recalcitrance, like women with artificial dentures. I thought to myself that there was really no need for her to do that.—She then opened her mouth properly and on the right I found a big white patch; at another place I saw extensive whitish gray scabs upon some remarkable curly structures which were evidently modeled on the turbinal bones of the nose.—I at once called in Dr M., and he repeated the examination and confirmed it... Dr M. looked quite different from usual; he was very pale, he walked with a limp and his chin was clean-shaven... My friend Otto was now standing beside her as well, and my friend Leopold was percussing her through her bodice and saying: ‘She has a dull area low down on the left.’ He also indicated that a portion of the skin on her left shoulder was infiltrated. (I noticed this, just as he did, in spite of her dress.)... M. said: ‘There’s no doubt it’s an infection, but no matter; dysentery will supervene and the toxin will be eliminated.’... We were directly aware, too, of the origin of the infection. Not long before, when she was feeling unwell, my friend Otto had given her an injection of a preparation of propyl, propyls... propionic acid... trimethylamin (and I saw before me the formula for this printed in heavy type)... Injections of this sort ought not to be given so thoughtlessly... And probably the syringe had not been clean (Freud, 1900, p. 107).

Freud’s interprets this dream as a wishful revenge dream: Freud was anxious to not be responsible for Irma’s unsolved pains, and the dream initially attributes the fault to Irma, before then reflecting Freud’s hope that the illness had an organic basis to absolve Freud of any blame for the lack of therapeutic success. By the end of the dream, it is Otto—Freud’s accuser in waking life—whose thoughtlessness is to blame for the failure associated with Irma’s treatment (pp. 118).

In contradiction to Freud’s interpretation, Gay (1988, p. 86) suggests that the dream was instigated by Freud’s doubts about his confidant Wilhelm Fliess surrounding the Emma Eckstein episode: “[t]he dream of Irma’s injection discloses, among other things, Freud’s anxiety to conceal his doubts about Fliess not just from Fliess but from himself.” Whether this is the case or not is, of course, pure conjecture, but it does raise the possibility that the dream itself expresses the *outcome* of repression. For example, the dream might defend Freud against the possibility of seeing his own failings via displacing blame, such that the unpleasure associated with the self-reproach prevents the self-reproach from being acknowledged (i.e., repressed, or projected, according to Hopkins, 2015 ^5^). Taken as such, the dream of Irma’s injection then reflects both Freud’s blindness of the seeing eye and the fulfillment of his wishes (the anxious denial of knowledge of being at fault). While this is all naturally speculative and open to various criticisms (see Anspaugh, 1995), the point here is to simply illustrate how repression could logically contribute to dreaming, without recourse to a censor. Given that repression itself is a wishful activity (in the negating sense), there is no logical objection or inconsistency in describing such dreams as both wish-fulling and reflecting the outcomes of repression.

At the same time, this account of repression alone does not address how improbable and impossible events occur in dreams. However, as is generally recognized, Freud never meant for repression to solely address dream bizarreness. Freud, in fact, states that “the most striking psychological characteristic of the process of dreaming” is that thoughts in propositional form undergo transformation into images (and particularly, visual images), which underlies the creation of the *hallucinatory experience*: “a thought, and as a rule a thought of something that is wished for, is objectified in the dream, is represented as a scene, or, as it seems to us, is experienced” (Freud, 1900, p. 534). And again: “In the case of the dream-work it is clearly a matter of transforming the latent thoughts which are expressed in words into sensory images, mostly of a visual sort” (Freud, 1916–1917, p. 180). In other words, this role of metaphoric dream imagery indicates that there are processes other than ‘censorship’ contributing to the apparent bizarreness of dreams.

To summarize, for Freud, motivation is the key for explaining why someone dreams of *x* while ignoring *y*, and although there are internal consistencies related to ‘censorship’ these are not fatal objections to repression contributing to dreaming. An account of repression and ‘censorship’ in dreaming can be provided that does not entail a ‘censor of dreams’ and is consistent with Freud’s general drive-motivational position. Consequently, there need be no fundamental disagreement between Freud and Hobson with respect to repression contributing to dreams (cf. Hobson, 1988, 2014d).

## Hobson’S Protoconsciousness Hypothesis and Functional Design Error

The paper now turns to addressing motivation in Hobson’s dream theory. Hobson’s position has evolved over four decades and incorporates several developments during that time. The initial activation-synthesis hypothesis proposes that dreaming is instigated by chaotic pons activity during REM sleep. Dreaming is the forebrain’s synthesis and interpretation of this noisy phasic PGO spike activity (Hobson and McCarley, 1977; McCarley and Hobson, 1977). In other words, “dreaming is the subjective awareness of brain activation in sleep and … the sleep activation of the brain results in the synthesis of conscious elements (e.g., emotion, perception, and thinking)” (Hobson, 2014c, p. 32). The later AIM model extends this internal activation (A), by adding input-output gate control (I), and changes in brain chemical modulation (M) (Hobson, 2009, 2014c). Based on these brain state differences Hobson proposes that REM sleep and waking are qualitatively different mental processes with differing neurochemical foundations and differing operations. Hobson (2009, 2014b,c), for instance, proposes that diminished self-reflective awareness and self-monitoring in dreams is attributable to REM brain sleeping:

Cognitive processes such as memory, self-reflective awareness, insight and judgment are deficient in dream consciousness owing to the shift in balance between the aminergic system (which is dominant in waking but ineffective in REM) and the cholinergic system (which is suppressed in waking but unfettered in REM) (Hobson, 2014c, p. 42).

Similarly, the bizarre nature of dreams occurs due to random PGO brainstem activation, aminergic demodulation, and deactivation of the dorsolateral prefrontal cortex during REM dreaming sleep (Hobson and McCarley, 1977; Hobson, 2007, 2009, 2013, 2014d). As Hobson and Pace-Schott (1999) more generally write, dreams are “bizarre because of the loss of the organizing capacity of the brain, not because of an elaborate disguise mechanism that rids an internal stimulus of an unacceptable meaning …” (Hobson and Pace-Schott, 1999, p. 211; cf, Hobson, 2009, 2014d).

More recently, Hobson (2009, 2013, 2014a,b,c,d) has proposed a protoconsciousness hypothesis whereby REM sleep dreaming generates “a state of consciousness that is a fundamental building block of waking consciousness” (Hobson, 2013, p. 162), important for both higher brain function and general physiological functioning such as thermoregulation (Hobson, 2009, 2013, 2015). REM sleep has a multifaceted integrative function: “[d]ream consciousness guarantees the binding of sense of self, motility, sensation and emotion. It is upon this base that waking consciousness is built” (Hobson, 2014a, p. 5).

Hobson rarely uses the term ‘motivation’ and his aim has obviously not been to develop a theory of motivation in dreaming. Consequently, we must piece together a possible role for motivational states within his theory of dreams. According to the initial activation-synthesis model, dreams were “motivationally neutral” (McCarley and Hobson, 1977, p. 1219) and reflected “value free sensorimotor dream stimuli” (Hobson and McCarley, 1977, p. 1336). In later writings, however, something akin to motivation is implicated in the role of endogenous instinctual acts, even if Hobson places the emphasis on ‘motor commands’ (Solms, 2013a). For example, Hobson (1988) writes that dreams play out endogenous ‘instinctual acts’ such as fear, aggression, defense/attack, approach/avoidance and sex (the “so-called four F’s of fixed actions”—“feeding, fighting, fleeing, and fornication”— Hobson, 1988, p. 295). These instinctual activities bear some semblance to the Freudian position where ‘behavior’ reflects elaborated fixed reflex patterns (“reflex processes become elaborated and coordinated into organized behavior”—Hobson, 1988, p. 294) (see Freud, 1900; see also Maze, 1983 for extensive discussion of innate consummatory activities becoming elaborated and modified through experience) ^6^.

Hobson (1988) also refers to ‘behavior-rehearsal’ theory in dreaming, indicating that there is an orderly relationship between instinctual actions and the content of dreams. Dreams are concerned with “important interactions” (Hobson, 2013, p. 160) and dreams provide an opportunity to rehearse skills in preparation for waking states:

Even if there are no outward signs of movement in REM sleep, motor programs in the upper brain are churning away. Internal networks can be checked while we sleep peacefully and have only slight recollections of this iterative process when we wake up..... Not only are we able to practice our skills while we sleep, but we might be able to produce and improve those skills in a safe behavioral vacuum.

This is indirect but strong evidence for the protoconsciousness hypothesis. Not only is the dreaming brain prepared to act in waking but it is prepared to act in an effective way. This is more than scenario framing, it is action enhancing! I don’t need to be conscious of how to ride a bike. My brain just knows how to do so because it has run my bike riding programs while I was asleep! (Hobson, 2014b, p. 18).

Rehearsing actions while asleep has an obvious survival advantage and one would expect that such important interactions to have been shaped by evolutionary pressures associated with survival and reproduction. The orderly nature of the dreaming process is further reflected in the “the selective activation of … important survival emotions in REM sleep” (Hobson, 2014c, p. 40). In this respect, Hobson (2013, p. 149) views emotions “as instincts with an important neurocognitive function,”, and he links emotions with instinctive responses that are necessary for survival:

The brain-mind is prepared to respond with fright, flight, and fight (or sometimes with approach) when stimuli evoke one or the other set of responses in waking. We are driven away or drawn closer to others by environmental signals and we already know instinctively how to respond to those signals (Hobson, 2014c, p. 40).

The survival-oriented, orderly nature of dreaming is further evidenced by the emotions typically found in dreams, such an anxiety and anger, which are primarily adaptive: “Anxiety is a signal, too, but a signal to the self, not the other. It is a warning signal and a very useful once. One should be anxious when walking alone on a dark street; survival may depend on it” (Hobson, 2013, p. 150). And again:

Protoconsciousness theory regards anxiety as an important attribute of consciousness. On Darwinian grounds, it makes sense for organisms to be wary, to be skittish, and to be ready to run away. If they did not flee, they might quickly be someone else’s lunch! Anxiety can, when exaggerated or amplified, become a symptom and it may even require treatment. But anxiety aids survival. It is thus existential and a strong guide to waking consciousness. Its presence in dreams is testimony to its importance in waking (Hobson, 2014d, p. 70).

Consequently, there appears to be no *prima facie* fundamental disagreement between Freud and Hobson here since instinctive activities such as fright, flight, fight, and approach presumably all entail an account of motivational drives (‘human nature’) to determine what we wish to approach and avoid, and so on (Maze, 1983; Boag, 2008, 2017). What is also noteworthy here is a possible convergence between Hobson’s position and affective neuroscience accounts, the latter of which similarly posit evolutionarily shaped emotional systems and their associated functional responses (Panksepp, 1999; Panksepp and Biven, 2012). As noted earlier, affective neuroscience research links drives and emotional systems with cognitive states (e.g., Watt, 2012, p. 100; Wright and Panksepp, 2012, p. 24), and given the SEEKING system’s involvement in dreaming and its instigation of goal-seeking behaviors and appetitive interactions with the world (Panksepp, 1999; Panksepp and Biven, 2012; Wright and Panksepp, 2012), Hobson’s (2013) appeals to ‘emotions’ here might possibly help provide his dream account with a motivational foundation that is not incongruent with the Freudian position.

Hobson’s protoconsciousness theory further proposes that REM sleep dreaming provides a primitive innate virtual reality generator, which enhances sensorimotor integration and real-world anticipation and prediction, even if unconstrained by the dictates of reality (Hobson, 2009): “REM sleep activates a virtual reality model of the world that is used in the construction of consciousness… [and] allows the conscious subject to anticipate and review important interactions between the self (EGO) and its environmental context” (Hobson, 2013, p. 160). Hobson further equates his virtual reality model with the ‘free energy’ model (Hobson and Friston, 2014)^7^ and Bayesian brain accounts, emphasizing how the brain essentially acts as an adaptive inference machine employing Bayesian probability to minimize ‘free energy’ (prediction error or ‘surprise’) (Friston et al., 2006; Friston, 2009, 2010; Carhart-Harris and Friston, 2010; Hobson and Friston, 2012, 2014). According to the free energy account, the brain minimizes free-energy by employing top-down expectations (internal hierarchical models) to predict sensory input and thus minimize prediction error. Such brain functioning is adaptive since “minimizing surprise allows biological systems to navigate the world in an orderly and predictable way” (Hobson and Friston, 2012, p. 87). REM sleep is also posited to play a particular role in minimizing free energy. During REM sleep dreaming, the brain prevents sensory input, which in turn reduces the complexity of input accumulated during wakefulness and allows the brain to optimize itself by minimizing the statistical complexity of its model of the waking world (Hobson and Friston, 2012, p. 87). A fundamental purpose of sleep is to help optimize this generative model of the world for optimal learning and inference during wakefulness. As Hobson and Friston (2012, p. 83) write: “We imagine that sensory data are sampled during wakefulness so that the brain’s model can be optimized or learned. Sleep corresponds to the process of *post hoc* model optimization that prunes redundancy and reduces complexity.” Accordingly, waking and REM sleep dreaming, while qualitatively difference processes, “both serve a common purpose—to optimize generative models of its world” (Hobson and Friston, 2014, p. 11). Bringing this altogether then, dreams involve instinctual fixed action patterns (‘foreordained scripts’), survival emotions, and predictive models that allow us to prepare for being awake. Dreams thus reflect a balance “between rehearsing what has already been learned about the world and exploring new hypotheses and possibilities that could be experienced” (Hobson and Friston, 2012, p. 95). This all suggests that REM sleep dreaming is orderly and functional, and the “evolutionary pressure to maintain dream consciousness during REM sleep—despite predation and thermoregulatory costs—speaks to the importance of actively maintaining a generative model of the world” (Hobson and Friston, 2014, p. 10).

## The Lawlessness of Dream Construction

While Hobson’s appeal to adaptive emotional instincts affords possible overlap with the Freudian position, an apparent problem for Hobson’s position is the continuing role of ‘lawlessness’ in his thinking (e.g., Hobson, 2009, 2013, 2014c). As described earlier, the activation-synthesis hypothesis proposes that dreaming is instigated by chaotic pons activity during REM sleep, which is responsible for dream bizarreness (e.g., Hobson, 2009). Hobson (2014d, p. 71) explicitly refers to the “lawlessness of dream construction” and believes that dream bizarreness is ‘explicable’ in terms of: “(1) the unavailability of the real world space-time continuum and (2) the chaotic nature of the REM sleep brain activation process” (p. 70). Hobson further believes that this position is justified based on quantum theory and the like:

Critics of the activation-synthesis hypothesis are troubled by the notion of chaos and are completely undone by the idea of randomness. For the strict Newtonian determinist (and Freud was certainly one of them), there is no such thing as an undetermined (or chaotic) event. Now, over 100 years later, quantum physics, chaos theory and the uncertainty principle are all enshrined as basic tenets of physics and chance is seen as an essential ingredient of all natural processes (Hobson, 2014d, p. 70).

Hobson (2014d, p. 70) even goes so far as to say that “[u]npredictability is the friend of dream scientists who cannot explain why a given dream plot was chosen on a given night.” Furthermore, he says, appreciating chaos provides a greater recognition of the brain’s complexity, as well as underscoring a place for humanism:

The brain is a physical object of such enormous complexity as to boggle the mind. But a moment’s reflection helps us to see that chaos and randomness are our allies, not our enemies. …. without chaos and without randomness, we are without freedom or, at least, the comforting illusion thereof. Here it suffices to say that the unpredictability that goes hand in hand with chaos, is the friend of creativity and novelty; it is also bail money for release from the otherwise inescapable jail of the repetition compulsion (Hobson, 2014d, p. 70).

However, whether ‘creative acts’ are best considered chaotic or not is not entirely clear since such acts do not appear random *per se* but instead both directed and orderly, and even explicable in terms of ‘desires and beliefs’ (see, for instance, Levine, 2015). Furthermore, there is a distinction between chaos and unpredictability (Boag, 2006b). Not being able to presently predict dream content does not necessitate an underlying chaotic process. There are presumably many orderly processes whose outcomes we are currently unable to predict, but nevertheless hope to do so in the future. Even infinite complexity does not rule out orderly causal relations occurring within systems; it simply makes prediction in any given instance more difficult, so that what may initially look chaotic may turn out to be orderly after all. On the other hand, proposing that dream contents are the result of ‘chaotic’ brain processes would mean that we are necessarily unable to explain why we dream what we dream.

Hobson, however, believes that randomness is a virtue of his position, and one that has functional justification. A random process, he says, is “welcomed as a guarantor of the thoroughness of whatever information generation process is at work. Any and all non-conscious information is a candidate for plot selection and appearance in dream scenarios as long as it is compatible with dream animation” (Hobson, 2014d, p. 71). Similarly, Hobson (2014b, p. 17) says that “chance plays a larger part in the shaping of our dream lives than we would like to admit. But the idea becomes more attractive when we recognize that a random process guarantees a more thorough check on the contents of memory than an overly determined mechanism would allow.” In other words, on Hobson’s account, randomness serves a function since any content would have equal chance of finding depiction in dreams.

One immediate problem with Hobson’s view about random dream plot selection is that the claim appears clearly falsified by the available evidence. If dream content selection was literally random, then all and any content would have equal chance of finding depiction in dreams. What we dream should then be like ‘an explosion in a paint shop’: very colorful, utterly disorderly, but never the same. However, this is by no means the case: dreams are coherent more often than not (Domhoff, 2005), and certain themes find expression more than others (Schredl et al., 2004). Moreover, recurring dreams should not occur under chaotic conditions when clearly they do (cf. Murkar et al., 2014). Furthermore, the frequency of drug dreams occurring during states of withdrawal also underscores the non-random nature of dream content, which, as discussed earlier, is explicable within a drive-motivational account (see Colace, 2014 for review).

On the other hand, Hobson’s appeal to chaos in dreaming appears to also undermine his later functional approach. As mentioned earlier, Hobson’s protoconsciousness hypothesis proposes that REM sleep dreaming involves “the selective activation of important survival emotions” (Hobson, 2014c, p. 40), as well as allowing enactments of ‘important interactions’ and ‘foreordained scripts’ (Hobson, 2009, 2013, 2014d). If so, then some elements of dreaming are adaptive and orderly. However, how this then fits with the “lawlessness of dream construction” (Hobson, 2014d, p. 71) is not entirely clear. Add to this that Hobson’s model “places the emphasis on design error and functional imbalance, rather than intrinsic conflict between instinctual and social forces” (Hobson, 2013, p. 152) and the situation becomes more confusing: both ‘lawlessness’ and ‘design error’ would appear to undermine the claim that dreams fulfill the orderly rehearsal of ‘important interactions’ (etc.).

Hobson’s broader account of lawlessness is also *prima facie* inconsistent with the free energy model. Hobson and Friston (2012, p. 87) write that “[t]he free energy principle is based upon the idea that biological systems resist a natural tendency to disorder,” and formulating and testing new models and hypotheses is presumably an orderly rather than chaotic process. Nevertheless, in justifying the lawlessness of the dream process, Hobson (2014d, p. 71) writes that “the activation-synthesis hypothesis is happy to applaud the lawlessness of dream construction because it takes that feature to be important to a view of the brain-mind as a constant novelty seeker and novelty creator. The brain-mind is not a plodding and repetitive automaton destined, forever, to loop around safe but uninteresting cerebral and ideational circuits.” More than this, though, Hobson writes that “our brains are as much creative artists as they are copy editors. What we may need to navigate our waking world is an infinite set of charts from which we may draw the one best suited to an equally infinite set of real-life possibilities (Hobson, 2009, p. 807; cf. Hobson, 2014d, p. 73). However, what mechanism could explain how a brain ‘chooses’ from infinite possibilities? Ignoring for the moment the impossibility of drawing literally from an infinite set of possibilities, without an orderly selection process, then the organism would be guided by non-sense rather than sense. While the free energy brain might formulate ‘novel’ hypotheses, it would be utterly inexpedient to base such predictions on a ‘careless,’ random process. Additionally, if the free energy model proposes that the brain both avoids ‘surprise’ and acts lawfully (so that modeling can occur) then there must be some orderly, predictable working of such a system. Furthermore, while it might be logically possible for a conjunction to occur between design error and functional outcome, given the available dream content evidence referred to above, it is more plausible to assume that distal shaping processes would make certain dream content more likely to occur than others given the evolutionary shaping of ‘important interactions,’ to use Hobson’s (2013, p. 26) terms (see Marsh and Boag, 2013). A more expedient position would be to link motivation and cognition, which provides a more compelling picture of how motivation contributes to the character of dreams.

To summarize, in the Freudian account, there is an orderly connection between motivational states and the content of dreams. According to Hobson, however, there is both something akin to an orderly relation between motivation contributing to dreams, but also the claim that the content of dreams is determined by chaotic processes unconnected to motivation. All in all, the functionality posited by Hobson’s protoconsciousness hypothesis simply does not sit squarely with the proposed lawlessness associated with the activation-synthesis position. While Hobson’s integration of affective systems is an advancement with respect to incorporating motivation into his theory of dreams, his appeal to lawlessness in dream construction appears both incompatible with aspects of his view that dreams provide functional support for consciousness, as well as contradicted by the available evidence.

## Conclusion

Given that both Freudian dream theory and Hobson admit that motivation contributes to dreaming, this paper examined the role of motivation in dreaming and assesses whether Freudian theory or Hobson’s alternative approach to dreams satisfactorily integrate motivation into their respective positions. Freudian theory provides an internally consistent general motivational account and proposes that motivation is the general key for explaining why someone dreams of *x* while ignoring *y* (even if other factors may be implicated). Nevertheless, Freud’s account of the teleological censor is untenable, although an alternative position addresses how repression could contribute to dreaming. On the other hand, Hobson’s position appears internally inconsistent since he proposes that dreams reflect orderly processes, providing environments for skill rehearsal and model development, while also stating that dream plot selection is random and based on design error and functional imbalance. However, Hobson’s appeal to emotions and instincts is a step in the right direction and provides a platform for approaching an understanding of the role of motivation in dreams, even if the specifics of the motivational systems require elucidation. While there may, of course, be alternative dream theories and explanations of the role of dopamine and motivation in dreaming, the findings suggest, if nothing else, that Freud was in the right direction, and it is up to future research to determine exactly how far his interpretation of dreams can be followed.

## Author Contributions

The author confirms being the sole contributor of this work and approved it for publication.

## Conflict of Interest Statement

The author declares that the research was conducted in the absence of any commercial or financial relationships that could be construed as a potential conflict of interest.

## Footnotes

1 The concept of explanation itself is complex, involving questions of reductionism and interactions between biological-neural properties and psychological acts (see de Jong, 2002, 2003). However, with respect to explaining human behavior, a natural science perspective entails addressing *antecedent* conditions that bring behavior about. These antecedent conditions can be thought about as efficient causes (the necessary and sufficient antedating conditions—Wise, 2004, p. 160). On the other hand, teleological accounts—whereby the effect or ‘purpose’ is construed as a cause of action—simply confuse effects with causes: “[t]he apparent goal direction of motivated behavior explains nothing; it is the mystery that remains to be explained” (Wise, 2004, p. 160).

2 Hobson (2013, p. 143) believes that Freud was an “inadvertent Cartesian,” and he holds Freud responsible for “the institutionalization of Cartesian dualism” that led to the disconnection between psychiatry and neurology. Hobson even believes that Freud eschewed the brain altogether: Freud “developed a speculative psychology with no relationship to brain science” (p. 143). In this respect, writes Hobson, Freud’s “denial of neurology leads to the unpardonable and intellectually impossible separation of mind and brain. This is the crypto-Cartesianism of which I speak critically. The mind does not operate on its own. It is not a separate entity from the brain. It has no set of laws that are all its own” (Hobson, 2013, p. 144). How Hobson comes to believe this is unclear. Hobson initially writes that he shares “Freud’s conviction that mind-body isomorphism is a valid approach” (Hobson and McCarley, 1977, p. 1335), which he considers “the most fundamental assumption of Freud” (McCarley and Hobson, 1977, p. 1211). Furthermore, Hobson (1988, pp. 60–65) extensively discusses Freud’s early theory of the brain in the *Project*. Be this as it may, while it is true that Freud chose to abandon his speculative neurology and discuss dreams on psychological grounds in the *Interpretation of Dreams* (1900), it was not due to the irrelevance of neuroscience but rather for practical purposes: as a methodology, *psycho*analytic investigations could only address psychological events. Indeed, Freud always considered the brain as an essential foundation (see Freud, 1913, 1940).

3 This is not to say, of course, that other factors do not contribute to any specific dream content. Presumably any event has a number of causal determinants, and the causal field also requires consideration. Nevertheless, on Freud’s view, motivation is a primary determinant of why we dream what we do.

4 Repression can also be compared with self-deception (e.g., Von Hippel and Trivers, 2011). For a discussion of logical difficulties associated with both repression and self-deception, as well as proposed solutions, see Boag (2007a, 2012, 2015a).

5 Hopkins (2015) writes that his interpretation of Freud’s dream of Irma’s injection with respect to projection is superior to Freud’s original wish-fulfilling one. While I do not disagree with Hopkins here, I simply point out that his projection interpretation is consonant with a general wish-fulfilling framework (a wish for something not to be the case).

6 Hobson (1988) also includes sleep as an instinct. However, Hobson (1988, 2013) also proposes that some behavior is ‘instinctual’ but other aspects not. For a critical discussion of this ‘ego-psychological’ position, (see Boag, 2014, 2017) and Maze (1983, 1987).

7 The free energy model has also been discussed in neuropsychoanalysis (e.g., Fotopoulou, 2013; Solms, 2013b; Hopkins, 2016), where Freud’s metapsychological economic position has been brought into relation to free-energy and hierarchical cortical systems (Carhart-Harris and Friston, 2010, 2012; Fotopoulou, 2013; Solms, 2013b; Hopkins, 2015). See Boag (2017), for a critical discussion of the general soundness of this account in terms of motivation.
